# Making Preference Assessments More Acceptable and Effective for People with Dementia

**DOI:** 10.1007/s40617-025-01145-x

**Published:** 2026-02-03

**Authors:** Lily Bigwood, Elin Staples, Rebecca A. Sharp

**Affiliations:** School of Psychology, The University of Auckland, Science Centre 302, 23 Symonds St, Auckland, 1010 New Zealand

**Keywords:** Dementia, Major neurocognitive disorder, Preference assessment, Social validity

## Abstract

For people with dementia, meaningful engagement with preferred activities is a vital component in maintaining quality of life. Preference assessments have been found to identify preferred and reinforcing stimuli for people with dementia. However, because they have been predominantly validated for individuals with intellectual or developmental disabilities, there are likely aspects of the assessment that need to be adapted. We implemented standard stimulus preference assessments for four people with dementia, and designed some adaptations based on objective measures of their choice, affect, verbal responding, and the input of the participants. We then compared the standard and the adapted versions in a multielement design. We found that both the standard and adapted assessments resulted in similar selections of stimuli. We observed an increase in indices of happiness and a decrease in indices of unhappiness for three out of four participants during the adapted version compared to the standard version. Family, staff, and the people with dementia reported both the standard and adapted preference assessments to be socially valid. One participant reported better understanding what they were being asked to do in the adapted assessment, and a staff member reported the adapted assessment to be more effective. We also found a decrease in the frequency of statements that were off-topic, socially polite, or about items being “sold” to them across all four participants in the adapted assessment. We further extended the work of Reid and Green (1996) by incorporating indices of confusion to capture a broader range of affect. On the basis of our results, we give some examples of how to make preference assessments more acceptable for people with dementia.

**Supplementary Information:**

The online version contains supplementary material available at https://doi.org/10.1007/s40617-025-01145-x.

Access to preferred activities is associated with improved quality of life in people living with dementia (Uceda-Portillo et al., 2024) (now called major neurocognitive disorder in the *Diagnostic and Statistical Manual of Mental Disorders*, 5th ed.; American Psychiatric Association (2013). A direct method of identifying preferred activities is through the use stimulus preference assessments. Preference assessments identify preferred items and activities by measuring choice among and engagement with several stimuli (Wagner et al., 2020). Typical direct measures of behavior during a preference assessment include approach, selection, and engagement with items in the array.

Preference assessments are used to identify items and activities likely to function as reinforcers (e.g., Quick et al., 2018) and have been used with individuals across various neurological diagnoses (e.g., Verriden & Roscoe, 2016) and vocal repertoires. There is a body of literature than demonstrates the utility of preference assessments for people with dementia. Researchers have observed that preference assessments conducted with people with dementia yield preferences that are stable and reflective of participant, not assessor, preference (Garcia et al., 2018; Mesman et al., 2011). Furthermore, stimuli identified via preference assessments have been used in interventions to increase responding related to the preferred stimuli. For example, Oleson and Baker (2014) conducted a free operant preference assessment in which participants were invited to interact with as many stimuli in the array for as long as they liked during the session. They taught participants to mand for materials required to engage in their preferred activities by withholding some of the materials and using textual prompts. Although the literature on the use of preference assessments for people with dementia is relatively small, the results of preference assessments can be used to identify preferred activities, to offer choice, and to identify potentially reinforcing stimuli (see Wagner at al., 2020, for a review).

However, researchers have also described unique difficulties in conducting preference assessments with individuals with dementia. For example, Raetz et al. (2013) described participant reports such as, “Why are you asking me again? I thought I just did this. I'm confused.” in response to the repeated presentation of stimulus arrays. Although the topographies of these reported behaviors differ across participants, they are perhaps a result of unfamiliar stimulus conditions (i.e., the way the preference assessment is presented). In the broader preference assessment literature (i.e., with other population groups), there have been studies that have demonstrated the effect of assessment variables and function on behavior. For example, Kang et al. (2011) found that for children with developmental disabilities, problematic behavior maintained by access to tangibles was evoked during assessments that involved removal of stimuli (e.g., paired stimulus), and problematic behavior maintained by attention was evoked during free operant assessments in which researcher attention was less available. However, problematic behavior was not evoked during preference assessments for the children whose behavior was maintained by escape. It could be that the function of the responses reported by Raetz et al. was escape (from the assessment), which contrasts Kang et al.’s findings that the demands to choose in an assessment did not evoke problematic behavior. Therefore, there is a potentially unique challenge in working with people with dementia not described in previous literature.

Wagner et al. (2020) suggested that for people with dementia and intact vocal-verbal repertoires, stimulus preference assessments might be aversive; they could simply tell the researcher or carers what they like. However, this is yet to be formally explored, and many studies report cognitive impairment rather than describing verbal repertoires. A more in-depth description of verbal repertoire may be useful in understanding social validity of the assessment, and therefore ways this may need to be adapted. Another impact of intact vocal-verbal repertoire on preference assessments was described by Oleson and Baker (2014), who added noncontingent attention from the researcher when the participant did not touch any of the items presented in the assessment, instead initiating interactions with the researcher. Therefore, there are a number of reasons for individualizing a preference assessment that a person with dementia receives, including considering their verbal repertoire.

An additional difference between individuals with dementia and participants included in the majority of previous preference assessment research is that older adults typically have far more established learning histories. An older adult is likely to have made contact with rules and contingencies for sharing with others, paying for unowned items, and taking items from strangers. For example, Lucock et al. (2020) reported that some participants were reluctant to make selections to avoid appearing “greedy” and that some thought they were helping with a taste test during preference assessments. Although these experiences are not unique to people with dementia, faulty stimulus control (i.e., that stimuli in the environment do not occasion the responses they used to) and deficits in remembering might compound their effect. For example, the person with dementia may not recognize that they are in a group home and do not have hosting responsibilities, or that they have met the implementer before. Therefore, while these issues in practice may arise with other populations, they may appear disproportionately when working with older adults.

It is clear that stimulus preference assessments can be effective in identifying preferred items for individuals living with dementia. However, there is little guidance on how to ensure the way these assessments, first developed and validated for use with other populations, are used is effective and acceptable for people with dementia. There is emerging evidence that these assessments may require adaptation to mitigate some of the specific issues a clinician or researcher might face in supporting someone with dementia. Additionally, we know of no studies that have evaluated the social validity of preference assessments for people with dementia using quantitative (e.g., indices of affect) or qualitative (e.g., surveys or verbal report) measures, neither for the people with dementia themselves nor carers.

Therefore, the aims of our study were threefold; to (a) add to the body of literature that shows that preference assessments can be used to identify preferred activities for people with dementia, including infrequently used single-stimulus and free operant assessments, (b) provide some examples of ways in which preference assessments can be framed or presented to mitigate the effect that the learning histories of older adults and social rules might have on the acceptability of how they are conducted, and (c) to measure the social validity of preference assessments for people with dementia (both qualitatively and quantitatively).

## Method

### Participants and Setting

Our participants were four older adults with dementia who were able to consent to their own participation; Anna (96-years-old), Luka (80-years-old), Nina (91-years-old), and Dorothy (96-years-old). Table 1 displays demographic data. All four participants had an intact vocal-verbal repertoire, which we defined as speaking in full sentences and responding accurately as a listener to other’s vocal-verbal responses. We did not formally assess participants’ cognitive repertoire, but identified whether each participant had mild or moderate cognitive impairment based on carer report. A participant was designated as having a mild cognitive impairment if they were largely independent in activities of daily living (ADL) such as getting dressed, bathing, and eating but required some input from staff (e.g., needed “reminders” or the environment to be augmented with written instructions), largely responded to environmental stimuli (such as someone talking to them), and was mostly accurate in identifying time and place. A participant was designated as having a moderate cognitive impairment if they were unable to complete ADLs without daily support from staff, required prompts to attend to environmental stimuli, required reminders to engage in activities, and inaccurately identified time and place.

**Table 1:** Participant demographic data

Participant	Age	Vocal-verbal repertoire	Mobility	Hearing	Vision	Cognitive impairment
Anna	96	Intact	Independent	Full	Normal	Moderate
Luka	80	Intact	Independent	Full	Normal	Mild
Nina	91	Intact	Used walker	Full	Mild impairment (distance)	Moderate
Dorothy	96	Intact	Independent	Hearing aide	Normal	Moderate

Anna, Nina, and Dorothy were identified as having a moderate cognitive impairment, and Luka a mild cognitive impairment. Luka did not view himself as having dementia, despite being diagnosed. All participants were ambulatory (Nina ambulated with a walker). Nina had a mild visual impairment (distance vision) and was unable to scan an array of items due to head mobility. None of the other participants had a visual impairment. Dorothy used hearing aids, but sometimes did not hear auditory stimuli when her hearing aides were not tuned in properly.

Anna, Luka, and Nina lived in Care Home 1, a large retirement village with a variety of care options (e.g., palliative care, respite care, individual care suites). Dorothy resided in Care Home 2, which offered independent living, assisted living, personalized care, and specialized dementia care. We conducted sessions in participants’ private living quarters.

#### Recruitment and Consent

Consent to recruit from within their service was gained from the care homes, after which a nominated representative from the service shared information about the study with potential participants. We met with interested residents of the care homes, provided additional information about the study, and gained written consent from willing participants. We used a supported decision-making process based on government guidance in New Zealand (Ministry of Social Development, n.d). Supported decision-making involved providing easy-to-understand information to the potential participant and a support person of their choosing, a meeting with a member of the research team to ask questions and gain clarification (e.g., about potential risks/benefits, data collection and storage/security, rights to withdraw), time to consider options. Once the person had chosen to participate, they and their support person communicated this in writing on a form that outlined who was present, their relationship with the person, a place for the person to consent in writing/record that they consented verbally, and who witnessed, recorded and agreed to this. It also included information on how the participant could withdraw their consent, and participants could indicate whether they wished to continue to participate if their ability to consent diminished during the study. Following consent, each participant was invited to nominate a family member and staff member for us to approach. We sought this person’s consent to participate (i.e., to provide social validity data; they were not asked to provide proxy consent for the person with dementia).

At the beginning of each session, we sought assent by re-introducing ourselves, stating that we were there to do research, what the purpose of the session was, and what we would be doing and recording. We recorded verbal assent if given vocally or non-vocally (e.g., a nod, “yes”). Prior to conducting sessions, we completed an adapted version of the Disability Distress Assessment Tool (DisDAT; Regnard et al., 2007) with family or staff carers to identify idiosyncratic behaviors that might indicate distress. The DisDAT has been validated for use in people with dementia and is clinically useful; however, psychometric properties are difficult to determine given that the behaviors identified are defined at an individual level (Jordan et al., 2012). The behaviors identified using the DisDAT would have resulted in termination of the session, but were never observed.

### Experimental Design

We used a 2-condition multi-element design (Bittle & Hake, 1977) to compare the standard preference assessments to an adapted version. For each participant, we conducted four sessions of the standard preference assessment to identify any adaptations in the implementation that may make the assessment more suitable. We selected this number to track whether indices of confusion might reduce with repeated exposure to the assessment. After the initial sessions, we alternated between the standard preference assessment (two sessions) and the adapted preference assessment (four sessions) in an order determined using a random number generator (random.org; Haahr, 2025).

### Procedure

#### Pre-assessment

We interviewed the participants, and either a staff member who knew them well (Luka, Anna, Nina) or a family member (Dorothy), to identify potential stimuli for stimulus preference assessments. We used the Pleasant Events Schedule–AD (PES-AD), (Teri & Logsdon, 1991) to structure the discussions. The PES-AD and the short version have reliability and validity with similar tools (Logsdon & Teri, 1997). We used items identified by both participants and carers in the preference assessments. Items included books, word searches, puzzles, etc.

#### Stimulus Preference Assessments

For each participant, we conducted four sessions of a stimulus preference assessment method as technologically described in existing literature. We chose the specific assessment used for each participant based on what we considered to be most appropriate for that client (i.e., based on their vocal-verbal repertoires and mobility). Luka and Dorothy could scan an array, were able to engage for longer sessions (i.e., more than 10 min), and enjoyed comparing items. Therefore, we selected a multiple-stimulus-without-replacement (MSWO) assessment for them. We selected a single-stimulus assessment for Nina because she was unable to scan an array and was unable to mobilize around the room without support. We selected a free operant assessment for Anna because she was able to mobilize independently, but would engage in high rates of conversation with the researchers that we predicted might preclude her from making a selection from an array. A free operant assessment allowed for a more “naturalistic” approach. At the beginning of each session, regardless of type of assessment, we provided 30-s access to each item in the array in a randomized order (i.e., sampling). We then began the assessment by saying “I’m going to show you some items you may like, you can interact with any of the items if you would like to, but you don’t have to. You can stop at any time.” If participants spoke to the researcher, staff member, or family member during the assessment, the person responded with a neutral statement such as “I see.” We did not initiate any conversation during the session.

A staff member (for Luka) was present for one session of the standard and one session of the adapted preference assessments so that they could comment on social validity. A family member (for Dorothy) was only available to comment on social validity for the standard assessment.

##### **Multiple-stimulus-without-replacement (MSWO)**

We conducted MSWO (DeLeon & Iwata, 1996) assessments for Dorothy and Luka. We placed six items equidistant from each other on a table in front of the participant and asked them to “choose one.” When the participant selected an item, they were given that item for 30 s and the others were removed from the table. After 30 s, we removed the item selected and re-presented the remaining items, rotating the array by taking the item that was furthest left and moving it to the furthest right. We continued this procedure until all six items had been selected. If the participant did not select an item within 30 s after being invited, we removed all of the array, waited for 30 s, and re-represented them rotated one place to the left.

##### **Free Operant**

For Anna, we placed seven items equidistant from each other on a table for 10 min (based on Roane et al., 1998). We invited her to “choose anything you like.” All items were available for the full 10 min.

##### Single-Stimulus

For Nina, we conducted a single-stimulus assessment of seven items based on Pace et al. (1985). We presented each item one at a time in a randomized order and asked “do you like this?”. If the participant engaged in a choice response (touched, pointed at, picked up, or asked for the item), we gave them access to the item for 30 s. After 30 s, we asked for the item back and presented the next. We continued this until Nina had been presented with all seven items.

#### Adapted Preference Assessments

For each adaptation, we describe the changed components below. All other aspects of the procedure remained the same.

##### **MSWO (Dorothy)**

We changed the verbal statement at the beginning of the session to “I have some items with me today. These items have already been bought, I did not buy them. You will not offend me or anyone if you do not like them and there is no judgement if you like them or not. We are not selling these items, we are just looking to see what kind of things you might like to do in your spare time.” We selected this adaptation because we noted a lack of correspondence between choice and engagement. For example, Dorothy chose the drawing pad and pens, but we noted these to be unused when we returned for the next session. We also noted Dorothy engaged in high rates of “social niceties” (e.g., offering tea or food), which may have indicated her selecting items to avoid offending us.

##### **MSWO (Luka)**

We changed the verbal statement at the beginning of the session to “These are not my items, so you will not offend me if you do not like them.” We also asked him after each choice why he had chosen/liked the item, and recorded his verbal explanation. We selected these adaptations because Luka suggested we should ask him why he selected each item to gather richer data, and because staff suggested to us that they often thought Luka would engage with items to please people, rather than because he liked the activity.

##### Free Operant (Anna)

Prior to each session, we provided Anna with 10 min of non-contingent social attention in the form of a casual conversation. We provided verbal statements or conversation-starters approximately every 30 s, and responded to all of Anna’s verbal responses and questions. We changed the verbal statement at the beginning of the session to “I hope you don’t mind that I have to quickly fill out these papers but while I am busy feel free to interact with any of the items available if you would like.” Anna engaged little with the items during the standard assessment, instead choosing to interact with the researcher. We provided 10-min of attention and verbal rule as the adaptation to reduce the establishing operation for social interaction and to signal that the researcher was unable to interact, and therefore increase the likelihood of her interacting with the items.

##### **Single-Stimulus (Nina)**

When Nina selected an item, we asked her why she liked it (and recorded her answer). If Nina initiated an interaction with us during the session (e.g., asked a question or made a comment), we responded with 1-min of social attention in the form of conversation. We adapted the preference assessment by increasing the social interactions we provided because Nina often stated that she was lonely when we offered her an activity. Providing social interaction allowed us to acknowledge this, while still assessing preference for activities.

No more than two sessions were conducted per day, with at least two hours between sessions. For Dorothy, sessions were 6 to 25 min in duration, 4 to 12 min in duration for Luka, 10 min in duration for Anna, and 4 to 8 min in duration for Nina.

### Measurement

We recorded continuous data on an iPhone 11 pro, 512GB with the free “Countee” app (Peić & Hernández, 2020) installed.

#### Choice and Engagement

In the multiple-stimulus-without-replacement (MSWO) assessment (Luka and Dorothy), we recorded choice responses, defined as touching, moving, picking up, pointing to, or vocally requesting an item or activity. In the free operant assessment (Anna), we measured the duration of engagement, defined as physical contact with the item. We recorded engagement with a 0-s onset and 0-s offset (i.e., the start of an occurrence was recorded as soon as the participant touched an item, and finished when they ceased contact). In the single-stimulus assessment (Nina), we measured the duration of engagement with items, as well as a verbal “yes” or “no,” or no response following the researcher asking, “do you like this?”.

#### Indices of Affect

We measured indices of happiness (IoH) and indices of unhappiness (IoU) based on the definitions in Green and Reid (1996) as continuous frequency data. IoH were defined as facial expressions that typically indicate happiness, including smiling, laughing, or vocalizations that indicate happiness (e.g., “hoorah!”). IoU were defined as facial expressions that typically indicate unhappiness, including frowning, grimacing, yelling while frowning, crying, walking away, and throwing objects. We also measured indices of confusion (IoC), defined as facial expressions that typically indicate confusion, including furrowed brow, raised eyebrow, blank stare, or vocalizations that indicate confusion (e.g., “huh?”).

We also recorded (by noting down the topography by hand, and recording the locus of time on “Countee”) any verbal statements that participants made during the assessments. A new occurrence of a statement was recorded when 3-s or more elapsed since the previous statement. We categorized these statements made by participants into four categories relevant to assessing the social validity of the assessment (either with regard to the purpose or method; Wolf, 1978). The five identified categories included on-topic statements about the items being sold (e.g. “Did you buy this?”), on-topic statements of social politeness or stimulus descriptions (e.g. “How lovely is this?”), off-topic questions to the researcher (e.g. “How is your family?”), off-topic statements (e.g., “I have three sons”), and off-topic statements about loneliness (e.g. “No one here talks to me.”). The first author developed the categories by reading all of the topographies of verbal statement and grouping them into themes. The first and third author then discussed the categories and agreed together on which statement fell into which category.

#### Social Validity

We gave each participant, and one staff member for Luka and one family member for Dorothy a social validity questionnaire (a) prior to preference assessment sessions, (b) after the standard preference assessments, and (c) after the adapted preference assessments. We provided a paper copy of the questionnaire and invited them to complete it without us present (we left the room and collected it later that day). We invited participants to ask a caregiver to help them complete it if they wished. Each question asked the respondent to rate statements on a 5-point Likert scale (1= strongly disagree, 3 = no opinion, and 5 = strongly agree). Before the preference assessments, we asked questions about boredom, the availability of activities, and whether staff knew the participants’ preferences to evaluate the social validity of conducting preference assessments. In the other two questionnaires, we asked about the acceptability of the procedure (e.g., the duration, the setting, whether they understood what was being asked). The participants were also welcomed to provide any additional comments at the end of the questionnaire.

### Interobserver Agreement and Procedural Integrity

A second, independent observer recorded data in 43% of sessions. For MSWO sessions, we calculated interobserver agreement (IOA) trial-by-trial; an agreement was scored if both observers recorded the same item was selected. We divided the number of trials in which both observers agreed by the total number of trials in that session and multiplied by 100. IOA was 100%.

For free operant and single-stimulus assessments, we calculated IOA by dividing the lower duration of engagement by the higher number of engagement for each item, and multiplied by 100. Mean IOA was 92% (median, 96%; range, 83% to 100%). Duration of engagement in absolute number of seconds ranged from 0 to 492 across sessions.

Because indices of affect and verbal statements were low-rate behavior, the IOA dataset was imbalanced (i.e., the behavior mostly did not occur). Therefore, on the basis of the recommendations by Cox et al. (2025), we calculated IOA using *F*1. We divided sessions in which a second observer collected data into 10-s bins. We summed the number of bins in which both observers recorded a response and divided it by the sum of bins in which both observers recorded a response and the number of bins in which the second observer recorded a response but the primary did not (the positive predictive value of the second observer; PPV). We calculated the true positive rate (TPR) of the second observer by dividing the number of bins in which both observers recorded a response by the sum of bins in which both observers recorded a response and the number of bins in which the primary observer recorded a response but the secondary did not. We calculated the *F*1 score by entering our PPV and TPR values into the equation provided by Cox et al. The closer the value to 1.0, the higher the agreement between observers. *F*1 for IoH was 0.9, for IoU was 1.0, for IoC was 0.87, and for verbal statements was 0.83.

An independent observer recorded procedural integrity data in 43% of sessions (across all types of preference assessment and participants). They used a procedural integrity checklist (task analysis of steps of the procedure) to record whether the researcher implemented each step correctly. Procedural integrity was calculated as the number of steps correctly implemented divided by the total number of steps and multiplied by 100. Mean procedural integrity was 99% (median, 100%; range, 88% to 100%).

## Results

### Identifying Preferences

We found that carers and the participants identified mostly the same stimuli (or classes of stimuli) as preferred by the participants (six of the seven stimuli identified by a participant was also identified by their carer, and Nina’s carer identified an additional stimulus that Nina did not). Verbally reported preferences largely matched preferred stimuli in the preference assessments; for three out of four participants, a highly preferred stimulus in the assessments was a preferred stimulus identified verbally (with some exceptions). Dorothy identified drawing and her carers identified drawing and arts and crafts as highly preferred. Her most preferred stimuli during the assessments were a puzzle, coloring, and Scrabble. Neither she nor her carers identified puzzles as most preferred when asked verbally. Luka identified reading and words, and his carers identified newspapers and cards. His most preferred stimuli during the assessments were newspaper, drawing, and word search. Cards (identified by his carers), were not highly preferred. Anna identified crosswords and newspapers, and her carers identified reading, crosswords, and magazines. Anna’s most highly preferred items during the assessments were books, magazines, and cards. Nina identified newspapers and yoga, and her carers identified magazines. Her most preferred stimuli during the assessments were book, yoga, and newspaper.

**Fig. 1:**
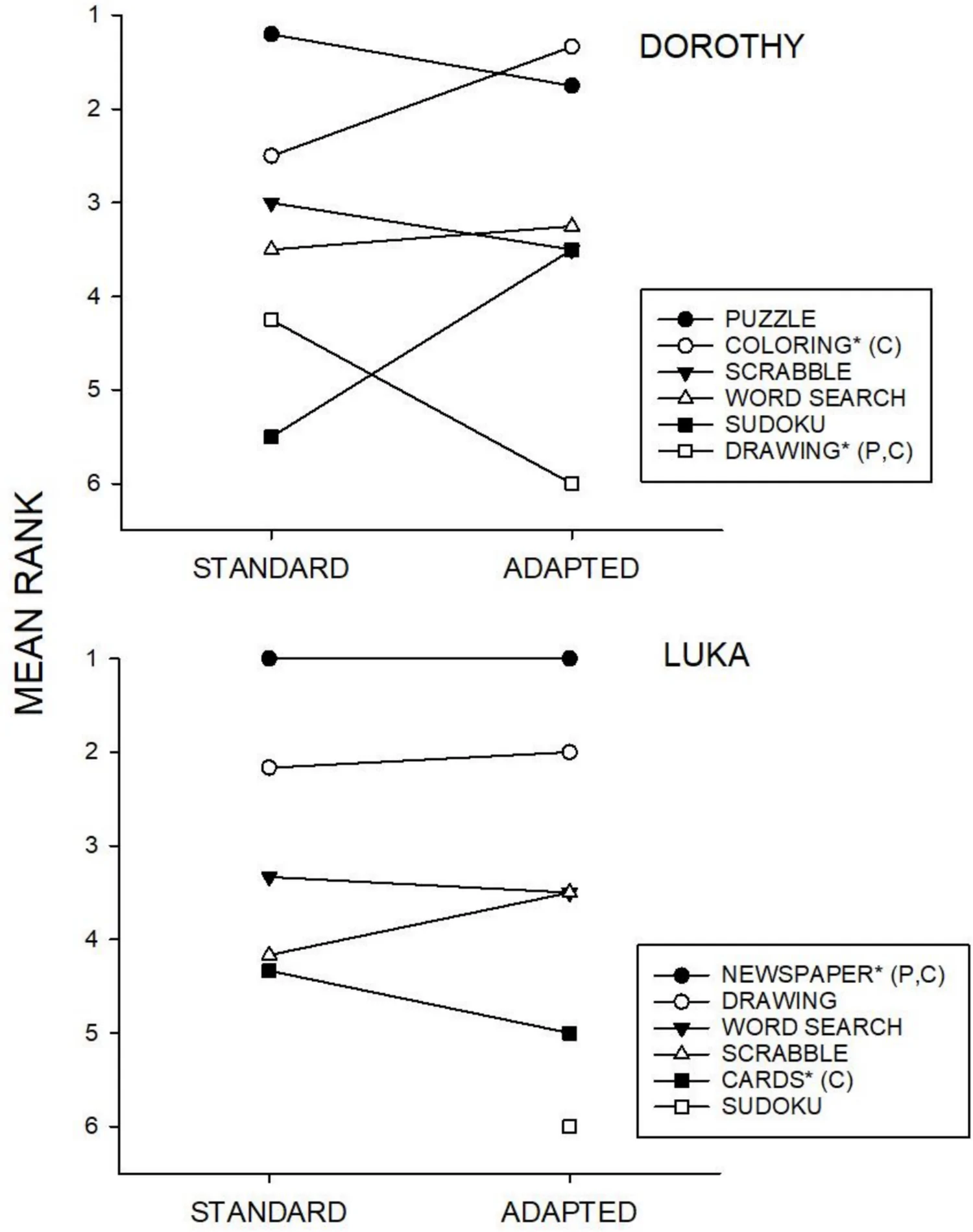
Mean rank of each item selected in standard and adapted MSWO preference assessments for Dorothy and Luka. A rank of 1 indicates the first item selected and 6 the last. Items identified as preferred verbally prior to the assessment by the participant (P) or their carer (C) are identified in the legend with an asterisk

**Fig. 2:**
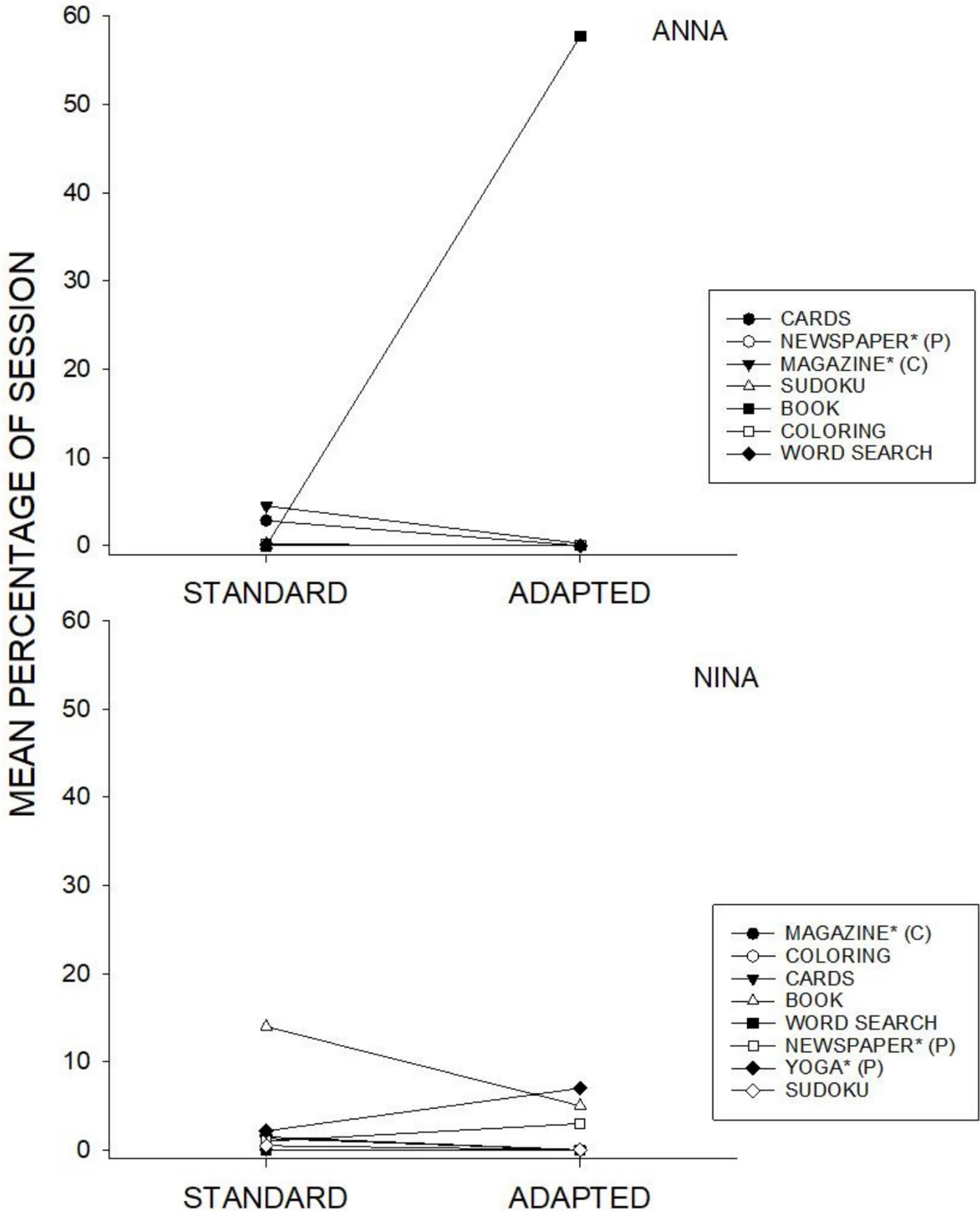
Mean percentage of standard and adapted preference assessment sessions in which Anna (free operant) and Nina (single-stimulus) engaged with each item. items identified as preferred verbally prior to the assessment by the participant (P) or their carer (C) are identified in the legend with an asterisk

For Dorothy and Luka, the rankings of items in the standard and adapted preference assessments were similar (Fig. 1), bar two exceptions. First, Luka never chose sudoku in the standard preference assessment, but did in the adapted assessment (albeit in a low ranking of 6th out of 6 items). Second, drawing was selected 3rd, 4^th^, or 5th in the standard assessment for Dorothy, but never during the adapted assessment (except for 6th, once). In the free operant assessment conducted with Anna (Fig. 2), she chose very few items and interacted with them for a very short duration during the standard assessment (interacted with cards, magazine for less than 5% of the session). In the adapted assessment, she interacted with the book almost exclusively (mean 58% of the session, range 4% to 82%), except for the magazine once very briefly. She did not engage with the book at all during the standard sessions. In the single-stimulus assessment, Nina interacted for a longer duration with two items in the adapted sessions than she did in the standard sessions (newspaper for a mean of 3% of the session, range 0% to 7%; and yoga for a mean of 7% of the session, range 0% to 10%) (Fig. 2). Regardless of session type, she engaged with items for a small percentage of the session (the highest percentage of engagement was observed for the book during a standard session; 34%, range 0% to 34%). She did not spend as much time engaging with the book during adapted sessions (mean 5% of the session, range 0% to 20%) compared to 14% (range, 0% to 34%) of the session for adapted sessions. She only engaged with the book in one adapted session out of four. Session-by-session data are available in the supplementary materials.

### Indices of Affect

**Fig. 3:**
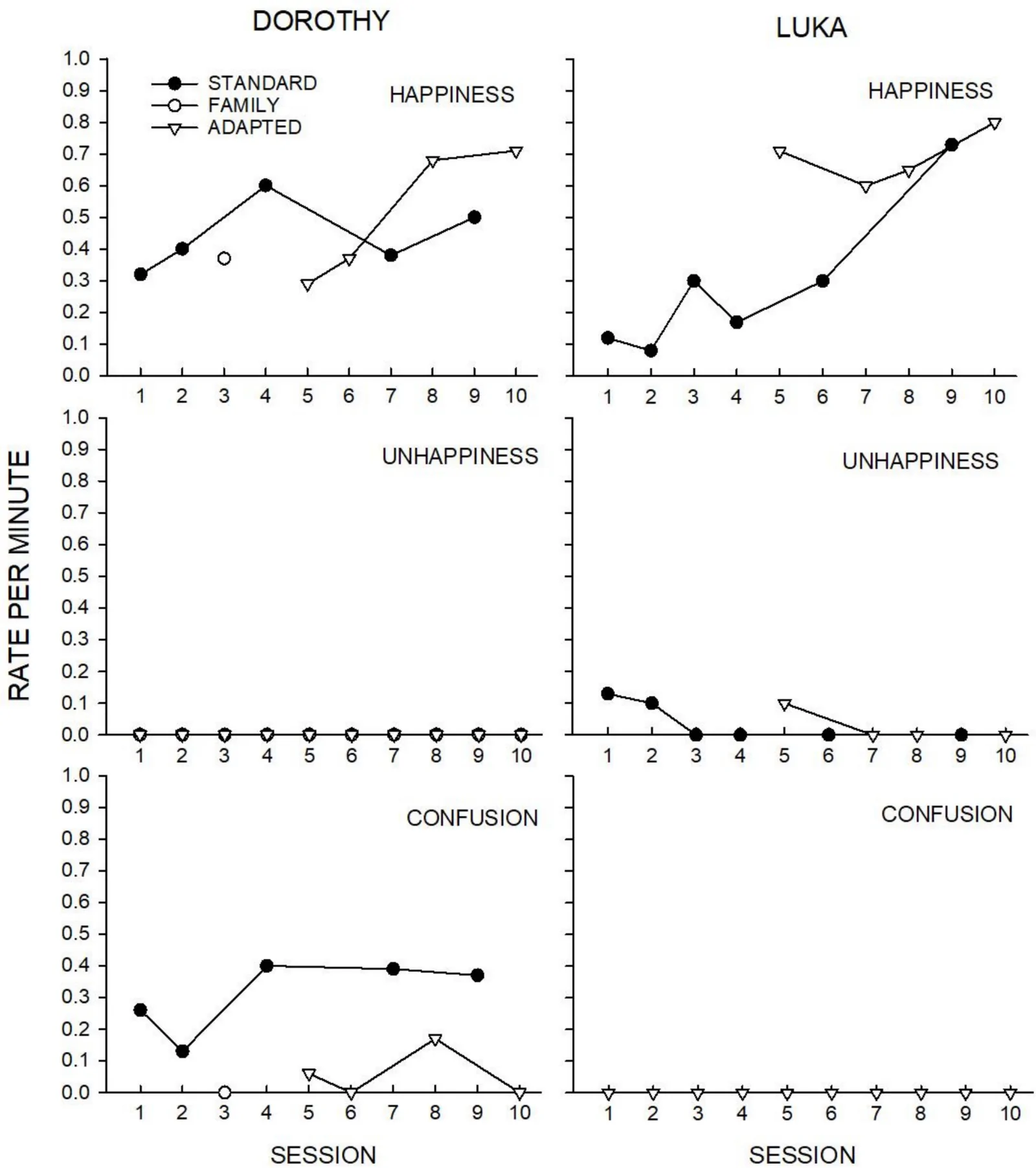
Rate of indices of affect (happiness, unhappiness, and confusion) per minute during standard and adapted MSWO preference assessments for Dorothy and Luka. Dorothy’s family was present during one of the standard sessions (depicted with an open circle)

**Fig. 4:**
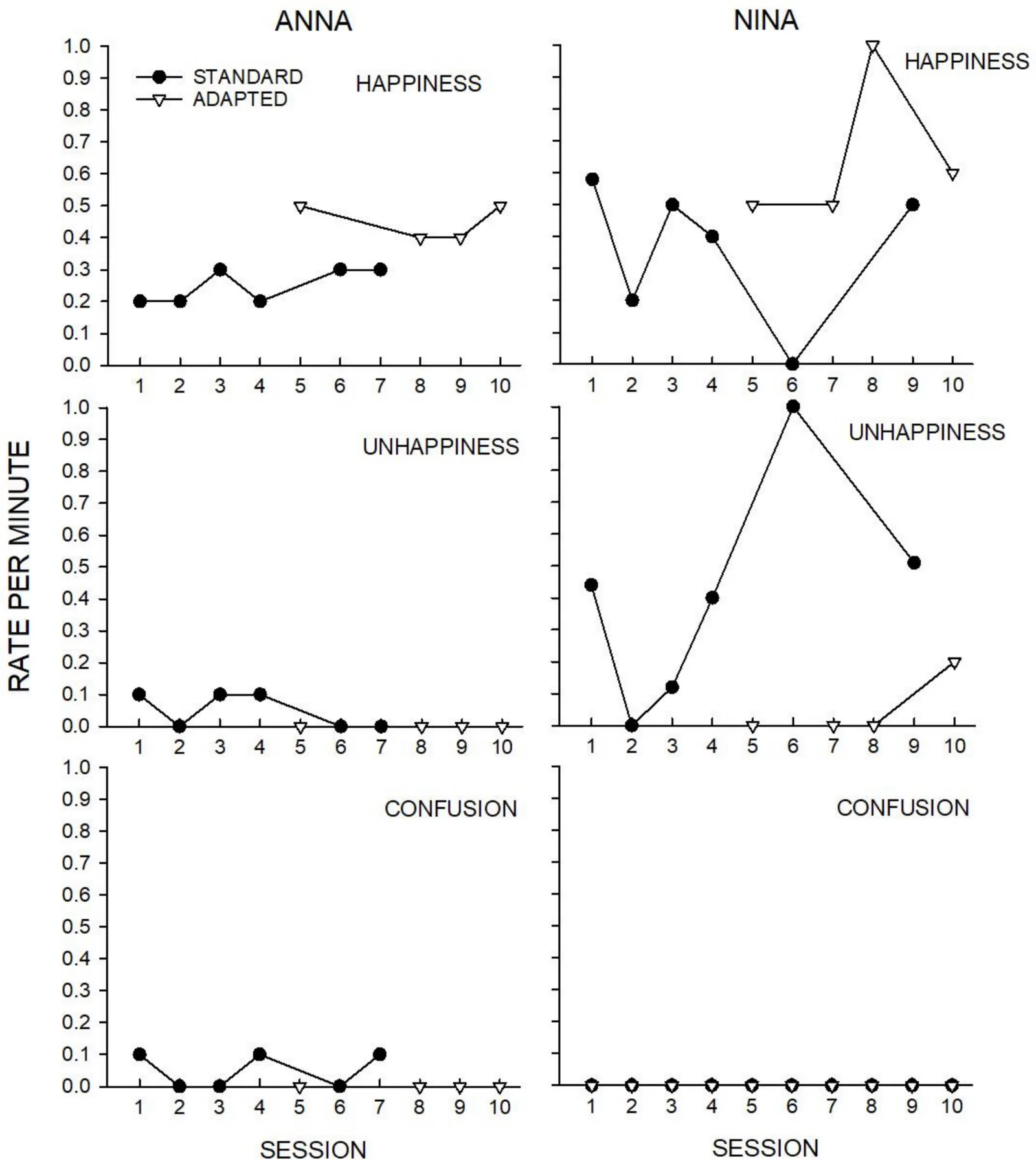
Rate of indices of affect (happiness, unhappiness, and confusion) per minute during standard and adapted preference assessments for Anna (free operant) and Nina (single-stimulus), respectively

**Fig. 5:**
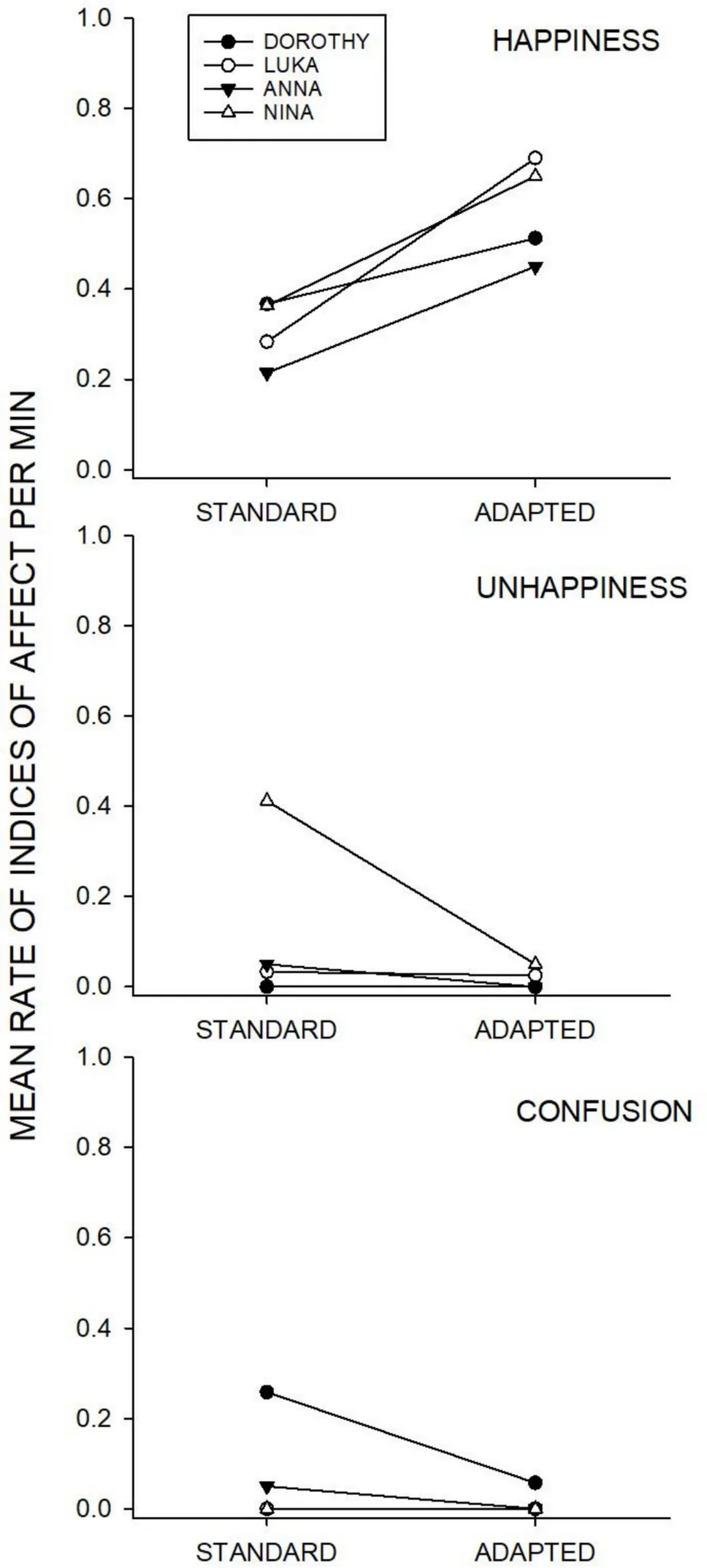
Mean rates of indices of affect (happiness, unhappiness, and confusion) per minute during standard and adapted preference assessments

Figures 3 and 4 show the indices of affect (happiness, unhappiness, and confusion) recorded for each participant in standard and adapted sessions, and Fig. 5 shows the mean rates across participants. For all four participants, higher rates of happiness (IoH) were observed in adapted sessions compared to standard sessions. For example, Anna’s IoH increased from between 0.2 and 0.3 per minute to between 0.4 and 0.5 per minute. Indices of unhappiness (IoU) were never observed for Dorothy (in either type of session). For Luka and Anna, we observed very low rates of IoU in standard sessions, and no IoU in adapted sessions (except for a very low rate, 0.1 per min, for Luka in the first adapted session). Nina engaged in more IoU in standard (range 0.2 to 1 per min) compared to adapted (0 to 0.2 per min) sessions. Neither Luka nor Nina displayed indices of confusion (IoC) in either type of session. Dorothy and Anna never displayed indices of confusion in adapted sessions, but both did during standard sessions (at a rate of 0.1 per min for both).

### Social Validity

With regard to statements about items being sold or for purchase, social politeness, off-topic conversation, and indications of loneliness or boredom, we found that all four participants emitted verbal statements that fell into these categories (Dorothy emitted statements that fell into three categories, Anna and Nina into two, and Luka into one) (Table 2). All of these verbal statements decreased in frequency when the adapted sessions were introduced with two exceptions. First, the frequency with which Dorothy engaged in “off-topic” statements did not change between the standard and adapted assessment. Second, Nina emitted more off-topic statements during the adapted sessions (i.e., mean of 1.5 compared to mean of 2.5 statements per session in the standard and adapted sessions, respectively). However, despite an increased mean, the range of frequencies was similar across standard and adapted sessions (between 1 and 3 occurrences, and 2 and 3 occurrences, respectively).

**Table 2:** Verbal statements made during standard and adapted sessions, categorized by theme. Session duration was 10 min for Anna, and ranged between 4 and 25 min for the other participants

Participant	Theme	Mean (range) statements per session		
		Standard	Adapted	Examples
Dorothy	Items being sold	2 (0–4)	0.3 (0–1)	“Did you buy these?” “How much are you selling those for?”
	On-topic social politeness	3.7 (3–5)	3.3 (2–4)	“Oh, this is beautiful!” “How lovely is this?”
	Off-topic questions	2.8 (2–4)	2.8 (2–4)	“How is your family?” “How are your studies going?”
Luka	Off-topic statements	1.3 (0–3)	1 (0–2)	Talked about his career
Anna	Items being sold	2 (1–3)	0.5 (0–1)	“I’ve already got that” “If I want that I can ask for it here.”
	Off-topic statements	5.8 (5–7)	4.5 (4–5)	Talks about childhood and family
Nina	Off-topic about loneliness	3.5 (2–5)	2.8 (2–4)	“This place is a mental desert” “No one here talks to me”
	Off-topic statements	1.5 (1–3)	2.5 (2–3)	Talked about childhood and career

We asked all our participants to fill out social validity surveys before any of the assessments, after the first standard assessment, and after the adapted version of the assessment. The answers for the participants with dementia are displayed in Tables 3 and 4. The answers to the initial before questionnaire were varied both within and between the individual participants. For example, Luka indicated that he had access to; found it easy to tell people; and that his support workers knew; the things he liked. However to the contrary, he also indicated being bored and not knowing a lot of things he liked to do. Moreover, Anna showed different results to some of the other participants and disagreed with wanting more activities to do in her care home, disagreed with often feeling bored, disagreed with not knowing many things she would like to do, and disagreed with her support workers knowing the things she like. On the other hand she agreed to finding it easy to tell people things she liked and agreed to having access to lots of things she liked. Participants responded similarly and positively to the questionnaires after both the standard and the adapted version of the preference assessment. One participant, Anna, politely declined answering the third questionnaire.

**Table 3:** Social validity responses from participants in the pre-assessment questionnaire

Pre-Assessment	Dorothy	Luka	Anna	Nina
I would like more activities to do	Strongly agree	Disagree	Disagree	Strongly agree
I often feel bored	Agree	Agree	Disagree	Strongly agree
I find it easy to tell people things that I like	Disagree	Agree	Agree	Agree
I have access to lots of things that I enjoy	Strongly agree	Agree	Agree	Strongly disagree
My carers know the things that I like	No opinion	Agree	Disagree	Agree
I don’t know many things that I like to do	Disagree	Disagree	Disagree	Disagree

**Table 4:** Social validity responses from participants after the standard preference assessment and after the adapted preference assessments

Pre-assessment	Dorothy		Luka		Anna		Nina	
	Standard	Adapted	Standard	Adapted	Standard	Adapted	Standard	Adapted
I enjoyed this assessment	Agree	Agree	Strongly agree	Strongly agree	Strongly agree	No answer	Strongly agree	Strongly agree
I liked the setting of the assessment	Agree	Agree	Agree	Agree	Strongly agree	No answer	Disagree	Disagree
I found this assessment difficult	Disagree	Disagree	Strongly disagree	Strongly disagree	No answer	No answer	Disagree	Disagree
The assessment was too long	Disagree	Disagree	Strongly disagree	Strongly disagree	No answer	No answer	Disagree	Disagree
I felt comfortable during this assessment	Agree	Agree	Strongly agree	Strongly agree	Strongly agree	No answer	Strongly agree	Strongly agree
I understood what the researcher was asking	Agree	Strongly agree	Strongly agree	Strongly agree	No answer	No answer	Agree	Agree
I think this assessment was helpful	Agree	Agree	Strongly agree	Strongly agree	No answer	No answer	Agree	Strongly agree

One staff member observed both the standard and adapted sessions for Luka. A family member observed a standard preference assessment for Dorothy (they were unavailable to observe an adapted session). The results of the social validity questionnaires completed by the family member and the staff member are displayed in Table 5. The family member reported to strongly agree with all questions, both before the first assessment and after the first assessment, and also provided an additional comment stating “I think the lack of stimulation could be a major problem for people with dementia.” The staff member mostly agreed and disagreed to all of the statements prior to any preference assessments. After the standard preference assessment, they strongly agree with all statements apart from one (to which they agreed). The staff member also added that they felt as if Luka was answering what he thought we wanted to hear. The staff member strongly agreed with all the statements following the adapted version, suggesting they thought this version was a more effective way of finding out what the person likes.

**Table 5:** Social validity responses from a family member and staff member in the pre-assessment questionnaire, after the standard preference assessment, and after the adapted preference assessments

Pre-assessment	Family member		Staff member	
I think that people with dementia benefit from more daily activities	Strongly agree		Strongly agree	
I think people with dementia experience boredom daily	Strongly agree		No opinion	
People with dementia often find it difficult to verbally communicate their wants and needs	Strongly agree		Strongly agree	
It is important people with dementia have easy access to things that they enjoy/like	Strongly agree		Strongly agree	
I think people with dementia would benefit from an assessment to specifically identify what they like	Strongly agree		Agree	
Finding specifically what people with dementia like will improve quality of life	Strongly agree		Agree	
	Standard	Adapted	Standard	Adapted
This preference assessment was an effective way of finding out what the person likes	Strongly agree	-	Agree	Strongly agree
I think that the person was comfortable during this assessment	Strongly agree	-	Strongly agree	Strongly agree
Communication was clear between the researcher and the person	Strongly agree	-	Strongly agree	Strongly agree
The setting of the assessment was suitable for the person	Strongly agree	-	Strongly agree	Strongly agree
The research team involved social rules that were suitable for the person	Strongly agree	-	Strongly agree	Strongly agree
The results of this assessment will be useful in their everyday lives	Strongly agree	-	Strongly agree	Strongly agree

## Discussion

We successfully adapted and implemented socially valid preference assessments for people with dementia by adding social attention or changing the verbal explanation of the session. Although the standard preference assessments were useful to identify preferred activities, we found that minor adaptations impacted overt indices of happiness, unhappiness, or confusion for these people with dementia, without compromising the effectiveness of the assessment for identifying preferred activities. Our study contributes to the existing literature in the following ways: (a) it adds to the body of literature that shows that preference assessments can be used to identify preferred activities for people with dementia, including single-stimulus and free operant assessments, which are infrequently reported; (b) it provides some examples of ways in which preference assessments can be presented to mitigate the effect of learning histories and social rules, and (c) is the first study to measure the social validity of preference assessments for people with dementia (both qualitatively and quantitatively).

By combining direct measures of indices of affect and verbal statements, in addition to choice and engagement, we were able to identify factors affecting the social validity of conducting preference assessments with people with dementia. These included access to social attention, social politeness, and misunderstanding of the purpose. Anna was the only participant for whom the adaptations substantially affected the results of the preference assessment. However, our data are of value not only because it is important to disseminate what does and does not change behavior, but they show how small adaptations increased the social validity of preference assessments. Increasing social validity matters, regardless of whether adaptations affect the outcomes of preference assessment. Leif et al. (2024) identified a number of reasons why social validity is important, including acceptance of methods by consumers, wide-scale implementation, and closing the research-to-practice gap.

To our knowledge, no studies have evaluated the social validity of preference assessments for either people with dementia or the people who support them. Aligned with calls to expand our suite of methods to evaluate social validity (see Leif et al., 2024 for a discussion), we measured social validity using both direct measures of indices of affect and using qualitative questionnaires. By extending Green and Reid’s (1996) work to include indices of confusion, we were able to capture a broader range of affect (and therefore more quantitative measures of social validity). We suspect that we could validate this measure and expand its use as a quantitative measure of social validity. However, we do acknowledge the low rate of this behavior in our participants, and therefore modest changes in indices of affect across the adapted and standard assessments.

Our social validity data capture two aspects of Wolf’s (1978) categories of social validation; of the methods used, and of the assessment purpose. Caregivers identified that identifying preferred stimuli for people with dementia is socially valid. The social validity data obtained from participants painted a mixed picture; three reported boredom but only two of those indicated they would like more activities to do. Three indicated that they had access to preferred activities and two indicated that carers knew their preferred activities. Therefore, we suggest that a valuable avenue for research is to identify how the results of preference assessments might be integrated into care, and by whom. For example, we know of no studies that have taught care staff to implement preference assessments for people with dementia, although this has been explored with other population groups (see Leaf et al., 2020 for a review). It might be that preference assessments are more likely to be used to identify preferred activities than stimuli that can be provided as contingent reinforcers for older adults; we did not capture any views on for what the data could be used. Additionally, we recognize that there is an absence of data on how best to capture social validity of preference assessments verbally for people with dementia and our questions may not have captured all important aspects of social validity. For example, we asked participants to indicate how much they enjoyed the standard and the adapted assessment but did not ask them to directly compare them.

Table 6 provides examples of how we sought to individualize the assessments to increase social validity. Some examples may be broadly applicable across people and settings (e.g., sitting side-by-side), whereas others may be practical in some circumstances but not others (e.g., offering food to non-participants). We suggest that practitioners select adaptations based on their clinical judgement. Put another way, and to align with the conceptualization offered by Schwartz and Baer (1991), we have extrapolated beyond what we have found with direct consumers (the participants) to provide guidance to members of the immediate community (i.e., those who support people with dementia) and perhaps by extension, members of the extended community (i.e., behavior analysts who support people other than people with dementia). While our focus was on reported issues specific to people with dementia and preference assessments, our findings contribute broadly to our understanding of the social validity of the behavioral tactics we use. We suggest our study could be used to stimulate similar evaluations with other population groups.

**Table 6:** Possible barriers to implementing stimulus preference assessments and suggestions of adaptations for practitioners

Possible barrier	Example	Adaptations to consider	Example
Social politeness	Answering to avoid offending or to please implementer Repeated statements of politeness or reciprocation	Providing context in the framing Making environment more natural Include others in the assessment	Explicitly stating that you will not be offended Sitting side-by-side, conversational tone, build rapport prior to assessment Offer items / food to others in the setting as not to single person out
Motivating operations for attention overshadow preference	Engaging only with the implementer instead of the items Initiating off-topic conversation	Non-contingent social attention Contingent social attention	Begin session with a conversation, embed trials in conversation Expanding choice responses measured to include asking why an item is preferred
Misunderstanding of purpose of the assessment	Believes that implementer is selling them the items Does not understanding that a choice response is expected	Adapt framing and instruction Remove distracting stimuli, add discriminative stimuli to make verbal instruction more effective	Explicitly state that items do not require payment / who has paid Implementer appears busy and indicates they are busy

A limitation of our study, however, is how we selected assessment methods and adaptations. We demonstrated the utility of free operant and single-stimulus assessments that have been used infrequently for people with dementia in the literature (no studies using single-stimulus to our knowledge). Wagner et al. (2020) suggested that the free operant method might be useful when a quick assessment is desirable and has the advantage of an “in-built” engagement assessment (i.e., duration of engagement) additional to choice. A single-stimulus assessment offers the same benefits. Wagner et al. also offered a list of factors that might inform method selection (e.g., purpose of assessment, ability to scan an array, fatigue). Although we considered these factors in our selection, we did so based on our clinical judgement rather than systematically. Our method would have been strengthened by the use of a decision-making tool that would have allowed us to select adaptations (or reject them) based on the same factors for each participant. For example, we could have used Lill et al.’s (Stimulus Preference Assessment Decision-Making System; SPAD, 2021), although it was derived from data largely from studies including people with developmental disabilities and therefore requires validating for people with dementia. Combining a tool such as this with some of the issues specific to working with people with dementia as identified previously might allow for replications of our study with more stringent methodological control. This would also allow for individualization to be achieved without needing to first conduct a standard preference assessment.

A limitation of some of the activities we used is that the duration of access in a trial (MSWO and single-stimulus) was short (30 s) and may have precluded meaningful interaction with the item (e.g., to identify a word in a word search). We suggest that future research evaluate appropriate durations of access during stimulus preference assessments for activities likely to be found in care homes. For some people with dementia, the value of leisure activities might include social stimuli (Lucock et al., 2020). For example, doing puzzles may have been a family activity or the person may have been involved in a weekly bingo night. Additionally, a lack of opportunities for social interaction in care homes may create establishing operations for activities that include interaction. Oleson and Baker (2014) included attention from the experimenter in their preference assessment because one participant preferred to interact with the experimenter rather than the activity. For two of our participants, changing the procedure to incorporate social attention resulted in the identification of stimuli that were not identified in the standard assessment.

To further explore the impact of social interaction available during preference assessments, future studies could replicate Kanaman et al. (2022), who found that although participants preferred stimuli paired with social interaction, the same items were identified as preferred when they were presented alone or with interaction. Our data also suggest an important avenue for future research is to assess preferences for social stimuli. For example, in addition to preference for attention overshadowing preference for items for Nina and Anna, Luka’s selection of items appeared to be more reflective of his preference to discuss items than engage with them. First, we could have evaluated this further by recording engagement if he was left alone with selected items. Second, we could have included social stimuli in our assessments. For example, Kronfli et al. (2024) evaluated preferences for conversation topics using vocally mediated self-report, MSWO, and a conversation assessment in which a topic was restricted after a pre-determined duration for autistic youth with vocal-verbal repertoires. Given that social reinforcers might be highly preferred by some people with dementia, this is an important avenue for future research.

A further contribution of our study is the evaluation of preference assessments for people with relatively mild cognitive impairment and largely intact verbal repertoires. Unlike other research that has found caregiver-reported preferences to be inaccurate (e.g., Mesman et al., 2011), we found that carer and self-reported preferences largely aligned with items selected during the assessment. For example, Ford et al. (2022) compared MSWO rankings with caregiver report. They found mostly inconsistencies between them, particularly with regard to moderately ranked stimuli. With regard to asking the person with dementia to report their preferences vocally, some studies have used indirect assessments such as the Pleasant Events Schedule Alzheimer’s Disease (PES-AD; Teri & Logsdon, 1991). However, few studies have compared self-reported preferences to the results of stimulus preference assessments, or evaluated vocally mediated preference assessments for people with dementia. Tessing et al. (2006) found that vocally mediated preference assessments that provided the selected item after the choice better identified reinforcing stimuli than assessments that did not provide the item after a choice. Vocally mediated preference assessments might be quicker and easier to implement more often, and mitigate some of the potential social awkwardness of conducting stimulus preference assessments for older adults (e.g., Lucock et al., 2020) and warrant exploration.

Although we found consistencies between verbally reported preferences and selected stimuli, the preference assessments provided more information than we gained from simply asking (i.e., identified further preferred stimuli for some participants, provided a ranking). For Dorothy, the stimulus preference assessment identified a preferred activity that she (and her carers) did not identify when asked. This would suggest that stimulus preference assessments can be a valuable tool for people living with dementia irrespective of their cognitive and verbal abilities.

Wagner et al. (2020) found in their review of the literature in which preference assessments were implemented with people with dementia that only half of the articles they reviewed reported information on the participants’ verbal repertoires. Therefore, we suggest that the suitability of preference assessments for people with varying cognitive impairments and communication abilities requires further exploration, and that our data suggest that they may have utility for people with mild cognitive impairments and largely intact verbal repertoires. For example, our participant Luka, who had mild cognitive impairment and an intact vocal-verbal repertoire, may not be typically considered for a stimulus preference assessment. However, Luka demonstrated high levels of indices of happiness during the assessment, which further increased once we asked him explicitly why he was choosing stimuli and how we could make the assessment more effective for him.

In conclusion, we found that relatively modest adaptations involving verbal statements prior to asking a person to make a choice and including social interactions can make stimulus preference assessments more acceptable for people with dementia, irrespective of effectiveness (and may make them more effective). What increases effectiveness and acceptability for each person with dementia will vary; however, we provided some examples of ways to increase the social validity of preference assessments. Our study represents a step toward demonstrating ways to address barriers that have been previously anecdotally reported, and may contribute to continued efforts to use the science of behavior to improve quality of life for people with dementia.

## Supplementary Information

Below is the link to the electronic supplementary material.

Supplementary file1 (DOCX 1545 KB)

Supplementary file2 (DOCX 181 KB)

## Declarations

### Data Availability

The data supporting the findings of this study are available from the corresponding author upon reasonable request.

### Assent

We obtained assent from the participant prior to each session.

### Clinical Trial Registry

This study was registered with the Australian New Zealand Clinical Trials Registry (12624000226505)
