# Supplementary material for: Making Preference Assessments More Acceptable and Effective for People with Dementia
Source: Behav Anal Pract. 2026 Feb 3;19(3):1361–77. doi: 10.1007/s40617-025-01145-x (PMC13638805; doi:10.1007/s40617-025-01145-x)
Supplement: Supplementary file 1 [file 40617_2025_1145_MOESM1_ESM.docx]

**Supplementary Figure 1**

*Rank of Each Item Evaluated in Standard (Black Bars) and Adapted (Grey Bars) Across MSWO Preference Assessments for Dorothy. A Rank of 1 Indicates the first Item Selected and 0 Means the Item was Not Selected in That Session.*

**Supplementary Figure 2**

*Rank of Each Item Evaluated in Standard (Black Bars) and Adapted (Grey Bars) Across MSWO Preference Assessments for Luka. A Rank of 1 Indicates the first Item Selected and 0 Means the Item was Not Selected in That Session.*

**Supplementary Figure 3**

*Percentage of Session in Which Anna Engaged with Each Item in Standard (Black Bars) and Adapted (Grey Bars) Free Operant Preference Assessment Sessions*

**Supplementary Figure 4**

*Percentage of Session in Which Nina Engaged with Each Item in Standard (Black Bars) and Adapted (Grey Bars) Single-Stimulus Preference Assessment Sessions.*
