# Supplementary material for: Making Preference Assessments More Acceptable and Effective for People with Dementia
Source: Behav Anal Pract. 2026 Feb 3;19(3):1361–77. doi: 10.1007/s40617-025-01145-x (PMC13638805; doi:10.1007/s40617-025-01145-x)
Supplement: Supplementary file 2 [file 40617_2025_1145_MOESM2_ESM.docx]

**PARTICIPANT INFORMATION SHEET**

**(Participant)**

**Note:** If you have trouble understanding any of this, you can ask a support person to help you.

**Project Title:** Evaluating the effectiveness and acceptability of behavioral assessments for people living with dementia

My name is NAME and I am studying towards a DEGREE at INSTITUTION.

My Supervisor is NAME who is a QUALIFICATION.

**Invitation to participate**

We are looking at finding a useful way for people living with dementia to tell others things that they like. You have been chosen as a possible participant as you may find this useful and important in your everyday life We would love to find a better way to help you communicate items or activities you like, in order for you to have things you enjoy and like to do. We will do this by doing sessions with you in a chosen area in your care home.

**Your choice**

You can choose if you would like to participate in this project or not. We have asked your care home not to change how you are treated, whether you choose to be involved or not, or whether you start the project but choose not to finish it. We have also asked your care home to make sure that your relationships and treatment of care will remain the same, no matter your decision, although as researchers we cannot guarantee this. Even if you agree to participate, you can tell us you wish to stop at any time without telling us why. If you choose to stop, we will keep the data we have already collected because we will have already started to analyse it.

We will also ask a family member and a support staff to be involved. You can choose who this is. If your support staff and / or family member no longer want to be involved with the project, you can still take part.

**What you will be doing**

We will do some sessions to find out what items/activities you like. They will take about 20 minutes. We will give you the option of a few items and will ask you to choose which one you like the best. We will record which items you choose and also record your facial expressions, movements and things that you say. The number of sessions we do each week is up to you. We will talk about this before you start the sessions. We will do between two and 10 sessions in total. We are looking to see what we can change about how we ask you what you like to help you make easier choices about things that you like. To do this, we will ask you and some of the people that support you what they thought of our sessions. We will ask both you and your support people (staff and family) to each fill out a short survey before and after the sessions. Based on their feedback, we will make some changes and show you the updated way of asking you what you like to do.

**Where and for how long?**

These sessions will run for a maximum of 6 months. All sessions will be at a time and place that works for you.

**Benefits and risks**

This project is to help find a better way for you to choose things you like to do. We hope to find more exciting things you like to do for your everyday life. If you ever feel stressed during any session, you can ask us to stop, and we will do so straight away. If you get tired, you can tell us that you want to take a break at any time. We will also talk to you and your support person to check that you are ok and still want to participate. If throughout the study your condition and ability to consent worsens, but you wish to still participate, you can still continue.

We will aim to create a safe environment where you feel valued and your dignity is respected. We want for you to feel empowered throughout this process.

**Your information**

During the sessions we will be looking at the choices you make and also how happy you are. We will store your information in a safe place in LOCATION. We will keep this information for x years and then destroy it. Only the researchers will be able to access your information.

We will tell people about the project and this information may be published in a scientific journal. We will not use your name or your care home. Only the researchers will know this information.

If you wish to see the results from our project, you can ask for this on your consent form. We are also happy to meet in person or over zoom to discuss the project after it has finished. You can bring a support person, like a family member or support worker, to the meeting if you want to.

**Student helpers** We will have other psychology students help us collect information. The students will only attend some of the sessions. They will not keep any of the information they have collected. The students will have to sign an agreement that says they will not share any of your information.

**Contact us**

If you have any questions about the study you can contact us by phone or email. We are also happy to meet with you and talk to you about the study in person or over zoom. You can send us an email and we will set up a time to meet that suits you. You are welcome to bring your family or support worker with you.

**Contact Details**

Please feel free to contact any of the people listed below for further information or any concerns about the research.

This study has had ethical approval from IRB on DATE, reference:

**Consent Form (Participant)**

**Please tick to indicate you consent to the following:**

| I have read the Information Sheet, or have had it read to me in a language I understand, and I fully understand what it says. |  |
| --- | --- |
| I have been given enough time to think about participating. |  |
| I have had the opportunity to ask someone to help me ask questions and understand the study. |  |
| I have had acceptable answers to my questions |  |
| I understand what I’ve been asked to do (answer questions and make choices). |  |
| I consent to be asked what I like, and to answer the questionnaires. |  |
| I understand that taking part is my choice and I can choose to take a break, or stop any time without a reason. |  |
| I agree that I would like to continue to take part even if my condition and ability to consent worsens.  No  Yes |  |
| I understand that my information and taking part will be kept confidential. |  |
| I understand that if I withdraw from the study, the researchers will keep data already collected. |  |
| I know who to contact if I have any questions. |  |
| I understand that there will be a second researcher present sometimes. |  |

**Declaration by participant:** I hereby consent to take part in this study.

| Participant’s name | |
| --- | --- |
| Signature: | Date: |

**Declaration by member of research team:**

I have given a verbal explanation of the research project to the participant, and have answered the participant’s questions about it. I believe that the participant understands the study and has given informed consent to participate.

| Researcher’s name: | |
| --- | --- |
| Signature: | Date: |

**INFORMATION SHEET FOR SUPPORTED DECISION MAKING**

**Project Title:** Evaluating the effectiveness and acceptability of behavioral assessments for people living with dementia

This sheet contains information about the research project we will help the person you support to make an informed decision about whether they would like to participate in the research project. We will explain the research project to the person with you present. You will be able to help clarify any information and ensure that we are explaining the project in a way the person can understand. This will help support the person to do the following:

- Understand all the information in their **Participant Information Sheet.** This sheet contains more detailed information about the project so you can clarify anything you’re not sure of and answer any questions.
- Give **informed consent** to participate/not participate in the project. If they decide to participate, we will support them to do this by either:

1. Supporting them to provide **written consent** by signing the **Consent Form*.*** If written consent is provided, you must also record this in the relevant section on the **Documentation of Consent form**.
2. Supporting them to provide **oral consent** by telling you, us *and* at least one other witness that they agree to participate in this study. If oral consent is provided, you must record that this has occurred in the relevant section on the **Documentation of Consent form*.***

- Please complete the **Documentation of Consent** form as a formal record of who was present and provided support during the person's decision making.

**DOCUMENTATION OF CONSENT**

**The Participant Information Sheet and Consent Form for the participant were both explained to:**

(Name of person providing consent – the participant)

The person stated above has provided their **written/verbal consent (please circle one)** to take part in this study. This person understands that they can withdraw their consent at any time without giving a reason. If they decide to withdraw their consent during the research, the data collected up to that point will still be available for the researchers.

| The Participant Information Sheet and Consent Form were explained to the participant by: | |
| --- | --- |
|  | (Researcher name) |

| Please state who was present during the time of this decision making. | | | | |
| --- | --- | --- | --- | --- |
| a) |  |  |  |  |
|  | (Name of witness) | (Relationship to Participant/Role) | (Signature) | (Date) |
| b) |  |  |  |  |
|  | (Name of witness) | (Relationship to Participant/Role) | (Signature) | (Date) |

| If the consent was **written**, please fill this section out: | | |
| --- | --- | --- |
|  |  |  |
| (Name of Participant) | (Participant’s Signature) | (Date) |

| If the consent was **verbal**, please write down who witnessed, recorded, and agreed to this. | | | | |
| --- | --- | --- | --- | --- |
| a) |  |  |  |  |
|  | (Name of witness) | (Relationship to Participant/Role) | (Signature) | (Date) |
| b) |  |  |  |  |
|  | (Name of witness) | (Relationship to Participant/Role) | (Signature) | (Date) |
